# Post-Translational Modification of Lamins: Mechanisms and Functions

**DOI:** 10.3389/fcell.2022.864191

**Published:** 2022-05-17

**Authors:** Mingyue Zheng, Guoxiang Jin, Zhongjun Zhou

**Affiliations:** ^1^ Medical Research Center, Guangdong Provincial People’s Hospital, Guangdong Academy of Medical Sciences, Guangzhou, China; ^2^ School of Biomedical Sciences, The University of Hong Kong, Hong Kong SAR, China

**Keywords:** lamins, nucleus, post-translational modification, laminopathy, genome regulation

## Abstract

Lamins are the ancient type V intermediate filament proteins contributing to diverse biological functions, such as the maintenance of nuclear morphology, stabilization of chromatin architecture, regulation of cell cycle progression, regulation of spatial-temporal gene expressions, and transduction of mechano-signaling. Deregulation of lamins is associated with abnormal nuclear morphology and chromatin disorganization, leading to a variety of diseases such as laminopathy and premature aging, and might also play a role in cancer. Accumulating evidence indicates that lamins are functionally regulated by post-translational modifications (PTMs) including farnesylation, phosphorylation, acetylation, SUMOylation, methylation, ubiquitination, and O-GlcNAcylation that affect protein stabilization and the association with chromatin or associated proteins. The mechanisms by which these PTMs are modified and the relevant functionality become increasingly appreciated as understanding of these changes provides new insights into the molecular mechanisms underlying the laminopathies concerned and novel strategies for the management. In this review, we discussed a range of lamin PTMs and their roles in both physiological and pathological processes, as well as potential therapeutic strategies by targeting lamin PTMs.

## Introduction

**FIGURE 1:**
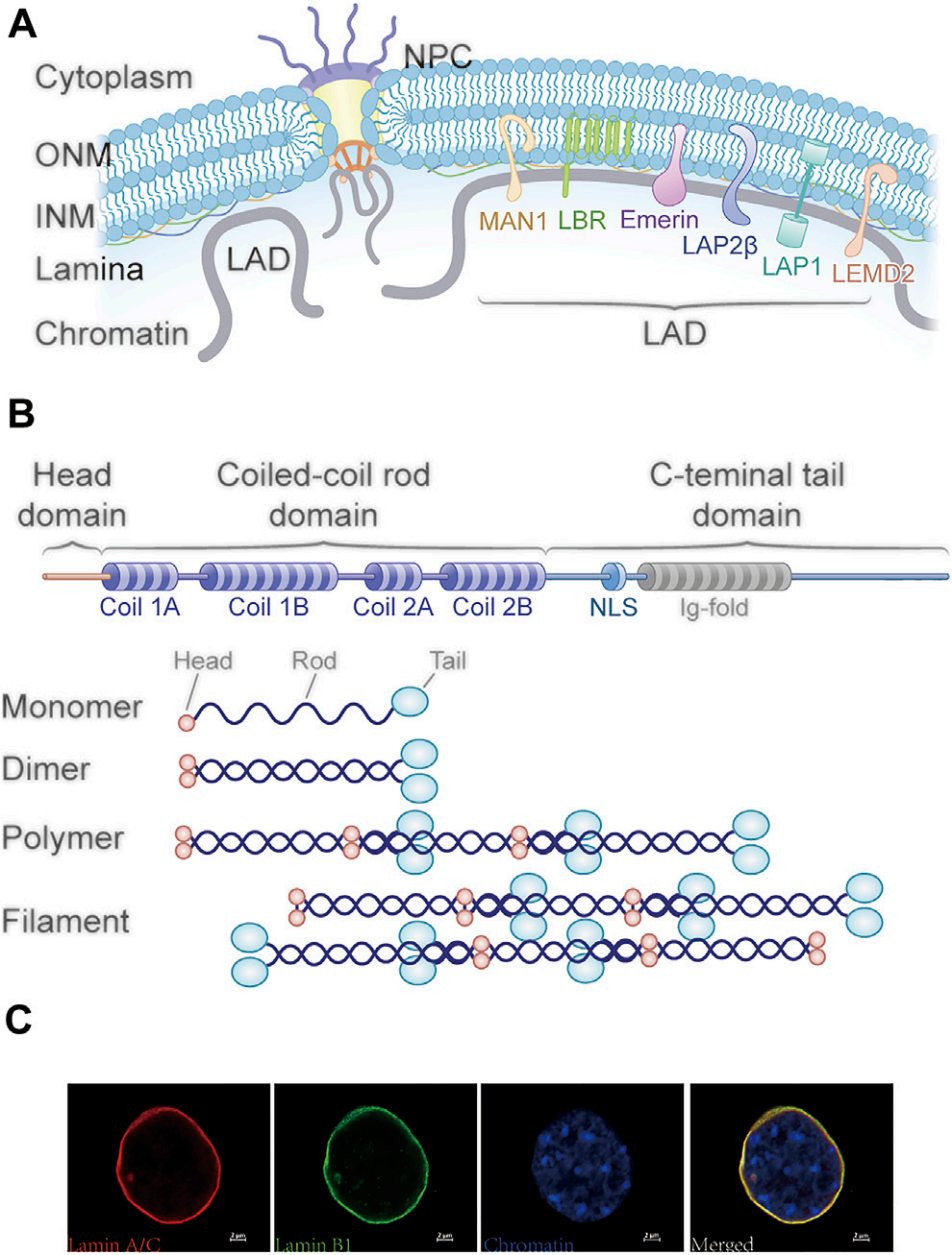
Structure of the nuclear periphery and lamins. **(A)** Schematic representation of the nuclear envelope structure. The nuclear lamina lies beneath the inner nuclear membrane and interacts with nuclear proteins and chromatin at lamina-associated domains (LADs). Several nuclear lamina proteins, including lamin A/C, B1, B2, lamin B receptor (LBR), and emerin, are associated with LAD positioning. **(B)** Schematic structure of lamins. The lamins consist of three domains including an N-terminal head domain, a central coiled-coil rod domain, and a globular C-terminal tail domain with the NLS and Ig-fold. Lamins assemble into higher order filaments through rod domain interactions. Lamin dimers form head-to-tail polymers and then assemble into filaments in an antiparallel manner. **(C)** Immunofluorescence images of mouse collecting duct cells stained for lamin A/C (red) and lamin B1 (green) and DNA (blue).

The nuclear lamina lies beneath the inner nuclear membrane and is a critical structure of the nuclear envelope. Apart from providing structural support for the envelope, it also interacts with nuclear proteins and chromatin at lamina-associated domains (LADs), therefore playing a critical role in genome organization and the architectural maintenance of chromatin (Gruenbaum and Foisner, 2015). In mammals, LADs are broad genomic regions with a median size of about 0.5 Mb and low transcriptional activity (Guelen et al., 2008; Peric-Hupkes et al., 2010). Several nuclear lamina proteins, including lamins (especially lamin B1) (Pickersgill et al., 2006; Guelen et al., 2008), lamin B receptor (LBR), and emerin (Figure 1A), have been shown to associate with the positioning of LADs (van Steensel and Belmont, 2017). Lamins, the major component of the nuclear lamina, are the type V intermediate filament proteins and exist in all metazoans, while being absent from unicellular organisms and plants (Cohen et al., 2001; Melcer et al., 2007). Accumulating evidence indicates that the lamin is a key player in maintaining the nuclear stability, nuclear and cytoskeletal organization, chromatin architecture, genome stability, nuclear assembly or disassembly, DNA replication and transcription, DNA damage repair, and differentiation and senescence (Hozak et al., 1995; Dorner et al., 2007; Lund and Collas, 2013; de Leeuw et al., 2018; Zheng et al., 2018). Liquid–liquid phase separation (LLPS) is an emerging mechanism of heterochromatin formation, and computational models are developed to disentangle the interplay of chromatin phase separation and lamina interactions during nuclear organization (Laghmach et al., 2020; Laghmach et al., 2021; Bajpai et al., 2021; Reynolds et al., 2021). For example, Reynolds et al. found that disruption of the nuclear envelope associated with lamin A/C depletion significantly increases the nuclear strain in regions of low DNA concentration using a mesoscale three-dimensional finite-element model of a cell nucleus (Reynolds et al., 2021). Another study showed the heterochromatin-lamina interactions in the nuclear organization enhance the mobility of euchromatin and indirectly introduce correlated motions of heterochromatin droplets (Laghmach et al., 2021).

Lamins include types A and B, encoded by different genes with significant sequence homology, similar structural features, and biochemical properties. Most vertebrates have only one type A lamin and two type B lamin genes, while *Xenopus* is a special animal model for the study of lamins, which possesses three B-type genes (Melcer et al., 2007). The *LMNA* gene encodes lamins A and C and the main A-type lamins, lamin A delta 10 and lamin C2, as well as a toxic isoform progerin/LA∆50, which will be described in detail in the following chapter (Lin and Worman, 1993; Nakajima and Abe, 1995; Machiels et al., 1996). Lamins B1 and B2 are the major B-type lamins, encoded by *LMNB1* and *LMNB2*, respectively. Besides, the latter also encodes another isoform lamin B3 (Furukawa and Hotta, 1993). A-type lamins are mainly expressed in differentiated cells but also found at extremely low levels in embryonic stem cells and inner cell mass of blastocysts (Eckersley-Maslin et al., 2013). In contrast, B-type lamins are considered to exist in all cells (Schatten et al., 1985; Lehner et al., 1987).

All lamins contain three domains: the N-terminal head domain, the central coiled-coil rod domain, and the globular C-terminal tail domain. The rod domain possesses four subdomains that mediate interactions with other lamina proteins. The C-terminal tail composed of a nuclear localization signal (NLS), an immunoglobulin-fold (IgG fold) domain, and a conserved CAAX motif (except for lamin C) mediates interaction with non-lamina proteins (Snider and Omary, 2014). Lamins first self-assemble into a coiled-coil dimer in a head-to-head manner in parallel association with two α-helical coiled-coil rod domains, and then, the lamin dimers form head-to-tail polymers; finally, they array in an antiparallel fashion into filaments (Figures 1B,C) (Guan et al., 2010; Zhao et al., 2010).

It has been reported that *LMNA* mutations and alterations in the lamin A/C expression or protein modifications drive a series of pathological progression (Bickmore, 2013; Briand and Collas, 2020). Mutations in the *LMNA* gene are the main cause of laminopathies, a spectrum of distinct genetic diseases attributable to mutations or altered post-translational processing of the nuclear envelope/lamina proteins (Maraldi et al., 2011). *LMNA* mutations are associated with diverse pathological conditions including muscular dystrophy (such as Emery–Dreifuss muscular dystrophy (EMD), limb-girdle muscular dystrophy 1B (LGMD1B) and dilated cardiomyopathy (DCM)), lipodystrophy, neuropathy, and progeroid syndromes (such as Hutchinson–Gilford progeria syndrome (HGPS) and restrictive dermopathy (RD)) (Dechat et al., 2008; Parnaik, 2008; Prokocimer et al., 2009; Dechat et al., 2010; Dittmer and Misteli, 2011; Parnaik et al., 2011; Ho and Lammerding, 2012; Burke and Stewart, 2013). In contrast to *LMNA*, few reports have associated human diseases with mutations in B-type lamin genes (Worman and Bonne, 2007), possibly due to the embryonic and perinatal requirement of B-type lamin genes (Kim and Zheng, 2013). Nonetheless, lamins B1 and B2 have also been implicated in genetic disorders affecting the heart, brain, and nervous system (Dauer and Worman, 2009).

The mechanisms underlying laminopathies have been extensively investigated. It is currently well recognized that lamins are functionally regulated by a myriad of PTMs, existing under certain biological or pathological conditions with specific functions, including farnesylation (Farnsworth et al., 1989), phosphorylation (Beausoleil et al., 2004; Olsen et al., 2006), acetylation (Choudhary et al., 2009; Lundby et al., 2012; Weinert et al., 2018), SUMOylation (Lumpkin et al., 2017), methylation (Rao et al., 2019), ubiquitination (Wagner et al., 2011; Povlsen et al., 2012), and O-GlcNAcylation (Alfaro et al., 2012; Wang et al., 2012; Simon et al., 2018). PTMs of lamins might directly affect their structure, therefore affecting the functions or destructing LAD binding or its organization and gene expression (Wong and Stewart, 2020), although the roles of different types and specific sites of PTMs remain to be further clarified. The different PTMs with corresponding mechanisms and their biological functions in association with human diseases are fundamental for us to target specific PTMs in lamins in the management of laminopathies.

## Post-Translational Modifications of Lamins

### Farnesylation and the Maturation of Lamins

**FIGURE 2:**
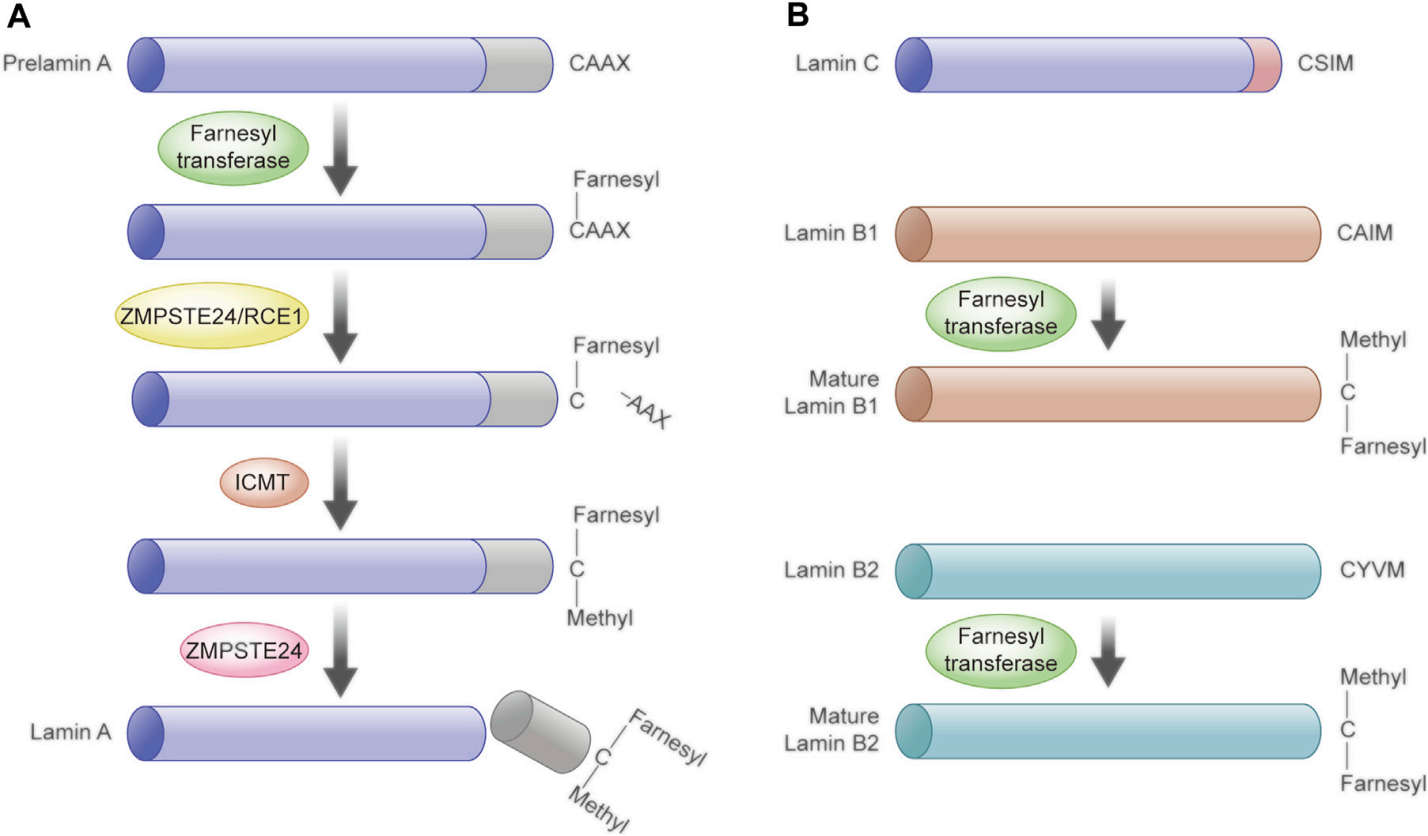
Post-translational procession of prelamin A, B1, and B2. **(A)** Post-translational procession of prelamin A to lamin A. Prelamin A undergoes four post-translational chemical reactions before the mature lamin A is formed. Step I, the farnesyltransferase mediates farnesylation of the CAAX motif of prelamin A. Step II, the last tripeptide is clipped off by ZMPSTE24 or RCE1. Step III, ICMT methylates the exposed farnesylated cysteine residue. Step IV, removal of the last 15 amino acid residues by ZMPSTE24, including the C-terminal farnesylcysteine methyl ester. **(B)** Lamin C does not undergo these processing steps as it does not contain the CAAX motif. The mature lamins B1 and B2 remain permanently farnesylated at their C-termini.

Farnesylation is critical for the biogenesis of lamins (Snider and Omary, 2014). Lamins with a terminal CAAX motif undergo farnesylation of the C-terminal cysteine with a farnesyl moiety by farnesyltransferase, including prelamin A (lamin A precursor) and lamins B1 and B2 (Liu et al., 2010). Prelamin A undergoes four post-translational chemical reactions to become fully matured lamin A (Young et al., 2005; Rusinol and Sinensky, 2006). In brief, aforementioned farnesylation of the CAAX motif is the first step that drives the clipping off of the last tripeptide by Ras-converting enzyme 1 (RCE1) or the zinc metalloproteinase Ste24 homolog (ZMPSTE24). Isoprenylcysteine carboxyl methyltransferase (ICMT) then methylates the exposed farnesylated cysteine residue, following the removal of the last 15 tail domain residues by ZMPSTE24, including the C-terminal farnesylcysteine methyl ester (Bergo et al., 2002; Pendas et al., 2002; Corrigan et al., 2005; Barrowman et al., 2008). Lamin C does not undergo this processing step because it does not have a CAAX motif, although being derived from the same gene with lamin A (Corrigan et al., 2005). In contrast, lamins B1 and B2 remain permanently farnesylated at their C-termini (Figure 2).

The farnesylated form of lamin A is associated with laminopathies. More than half of laminopathies are caused by *LMNA* gene mutations, in which HGPS is the thoroughly studied one that is caused by heterozygosity for LMNA point mutations, such as the canonical G608G mutation. The mutation leads to alternative pre-mRNA splicing and production of an irreversibly farnesylated form of truncated prelamin A, generally called progerin or LA∆50 which causes premature aging (Smallwood and Shackleton, 2010). Restrictive dermopathy (RD) is caused by the autosomal recessive gene defect of ZMPSTE24 or a homozygous classical mutation in the *LMNA* gene, resulting in an accumulation of a farnesylated and methylated prelamin A (Smallwood and Shackleton, 2010). Zmpste24 deficiency could increase DNA damage because of defective 53BP1 recruitment in Zmpste24^−/−^ mouse embryonic fibroblasts (MEFs) and in HGPS fibroblasts, resulting in delayed DNA damage repair and genomic instability (Liu et al., 2005). RD is a perinatal-lethal progeroid syndrome, but its diversified phenotypes depending on the site of the mutation contribute to the differences in the patient condition (Gruenbaum and Foisner, 2015). Disruption of lamin A maturation by ZMPSTE24-knockout mice and knock-in Lmna^G609G^ in mice recapitulates most of the premature aging phenotypes of the human diseases (Varela et al., 2005; Young et al., 2005; Liu et al., 2012; Ghosh et al., 2015).

On the other hand, the role of farnesylation of lamin B1 is also emerging. Farnesylation deficiency of lamin B1 or B2 was reported causing neuronal migration defects within the developing brain (Coffinier et al., 2010; Kim Y. et al., 2011; Coffinier et al., 2011; Hutchison, 2014). Another study reported that farnesylation of lamin B1 is essential for retaining chromatin within the bounds of the nuclear lamina during neuronal migration of brain development in knock-in mice expressing nonfarnesylated lamins B1 and B2 (Jung et al., 2013).

## Phosphorylation: Multifunctional Post-Translational Modifications

Phosphorylation is the most extensively studied and common PTM of lamins. It has been reported that their head and tail domains had a higher frequency of phosphorylation than the rod domain (Machowska et al., 2015). Lamin A possesses over 70 identified unique Ser/Thr phosphorylation sites (Simon and Wilson, 2013; Machowska et al., 2015), some of which have been revealed to be critical for its structural features and functions. The phosphorylation enzymes of lamins include Cdk1, protein kinase C (PKC) (Goss et al., 1994; Thompson and Fields, 1996; Collas et al., 1997; Collas, 1999), cellular kinases, mitogen-activated protein kinase (MAPK), protein kinase A (PKA), casein kinase II, and AKT (Gruenbaum et al., 2003; Gruenbaum et al., 2005; Margalit et al., 2005), in which Cdk1 phosphorylates the N-terminal Ser22 and the C-terminal Ser392 in human lamin A/C or analogous residues in B-type lamins, and PKC phosphorylates S22/S392 and S395/S405 sites of lamins, and S404 is the target of AKT. In addition, lamins also have a few tyrosine phosphorylation sites; the epidermal growth factor receptor (EGFR) was reported to phosphorylate lamin A at several tyrosine residues *in vitro*, including Y45, Y81, Y211, Y359, Y376, Y481, and Y646 (Tsai et al., 2015). Moreover, Scr has been found to be able to regulate the assembly of nuclear lamina by phosphorylating lamin A, especially at Tyr45 (Chu et al., 2021). Lamin functions depend on the capacity for polymerization and depolymerization, in which the phosphorylation contributes to the easily reversible regulation of their ability to form polymers and solubility. The site-specific phosphorylations of A-type lamins have been shown to influence their nuclear envelope inner layer targeting and then regulate lamin assembly or interaction with chromatin (Haas and Jost, 1993; Goss et al., 1994; Leukel and Jost, 1995). Phosphorylations of B-type lamins could also alter their assembly state or inhibit their nuclear envelope targeting (Hennekes et al., 1993; Hocevar et al., 1993; Goss et al., 1994). Thus, lamin phosphorylation is critical for nuclear assembly and chromatin organization and is involved in multiple nuclear functions.

### Phosphorylation and Cell Cycle Regulation

It has been confirmed that the phosphorylations at different residues of lamins play critical roles throughout the cell cycle. Disassembly of lamins is required for nuclear envelope breakdown in the early stage of mitosis. Plenty of studies revealed that this process was dominated by lamin phosphorylation under the catalysis of Cdk1 (Dessev et al., 1990; Heald and McKeon, 1990; Peter et al., 1990; Mall et al., 2012). Moreover, phosphorylated lamin A/C is released into the cytoplasm, while phosphorylated farnesylated B-type lamins remain membrane bound but disperse throughout the ER during mitosis (Gerace et al., 1984; Georgatos et al., 1997). Mitotic lamin phosphorylation sites are commonly called “mitotic sites,” which include Ser22 and Ser392 in lamin A/C, Ser23 and Ser393 in lamin B1 (amino acid position in UniProtKB P20700), and Thr34, Ser37, and/or Ser405 in lamin B2 (amino acid position in UniProtKB Q03252) (Heald and McKeon, 1990; Peter et al., 1990).

In contrast with mitosis, some studies found that phosphorylation of lamin A/C focused on the head, the beginning, and the end of tails in interphase cells. Some kinases, such as Cdk1 and PCK, can also phosphorylate lamins in the interphase; a few sites are reported as mitotic sites, such as Ser22 and Ser392, but with a much lower rate than mitosis. A new study reported that Ser22 phosphorylation of lamin A/C could modulate the cardiac sodium channel, associated with cardiac conduction disease (Olaopa et al., 2021). Another research demonstrated that lamin C was more strongly phosphorylated at Ser22 than lamin A in interphase fibroblasts (Kolb et al., 2011); the reason might be that lamin C was easy to be touched by kinase due to its proximity to the nuclear interior (Kolb et al., 2011). A recent study found abnormal phosphorylation of lamin A at Tyr45 caused the disassembly of lamina in interphase cells by Src (Chu et al., 2021). Ser390, Ser404, Thr424, and Ser652 residues of lamin A are also reported to be phosphorylated during the interphase (Swift et al., 2013). Thus, precise regulation of lamin phosphorylation is critical to nuclear structural integrity and genomic stability.

### Phosphorylation and Laminopathies

Lamin phosphorylation is closely associated with laminopathies. A new genotype-phenotype analysis predicted that pathogenic LMNA mutations were correlated with changes of lamin phosphorylation, especially those located in the head and tail domains by machine learning methods, and they predicted that phosphorylation of Y45 and Y481 is associated with laminopathies (Lin et al., 2020). Ser22 is a key phosphorylation site during mitosis, contributing to depolymerization of lamin A/C. Its phosphorylation of permanent farnesylated lamin A mutants is reported to be defected and associated with the pathogenesis of laminopathies (Osorio et al., 2012; Moiseeva et al., 2016; Liu and Ikegami, 2020). Recently, a study has investigated the relationship between Ser22 phosphorylation of lamin A/C and progeria mutations, as well as its possible mechanism (Ikegami et al., 2020). They found Ser22-phosphorylated lamin A/C was not only localized to the nuclear interior but was also able to bind to active enhancers in euchromatin in the nuclear interior in human fibroblasts throughout the cell cycle. However, a subset of Ser22-phosphorylated lamin A/C-binding sites was found to be lost in progeria-patient fibroblasts but emerged in normally quiescent loci; then, new Ser22-phosphorylated lamin A/C binding was accompanied by increased histone acetylation, increased c-Jun binding, and upregulation of nearby genes implicated in progeria pathophysiology.

Cho et al. found that Ser458 phosphorylation was only found in muscle biopsies from myopathy patients with mutations in the Ig-fold motif of A-type lamins and not found in those from control patients with any other neuromuscular diseases, and they further confirmed that phosphorylation of Ser458 was only found in lamin A mutants associated with myopathy, whereas not in lipodystrophy- or progeria-associated mutants. Furthermore, they revealed that this disease-specific phosphorylation of A-type lamins was induced by Akt1, and it contributes to myopathy caused by LMNA mutations (Cho et al., 2018).

It still remains to be clarified whether laminopathy mutations may directly alter the phosphorylation of lamins, or the change of lamin phosphorylation in laminopathies is a result of the mutation or a compensatory mechanism. Therefore, investigation into lamin phosphorylation in laminopathies is conducive to deeply understand how laminopathies arise and why they are so specific.

### Phosphorylation and Viral Infection

Reversible disassembly of the nuclear lamina through regulating phosphorylation facilitates the mitosis and nuclear export of large messenger ribonucleoprotein (mRNP) complexes and becomes a sharp blade for some virus achieving nuclear egress of capsids. For example, herpes simplex virus 1 (HSV-1), HSV-2, and HCMV infections can cause phosphorylation of all three types of lamins (Marschall et al., 2005; Park and Baines, 2006; Mou et al., 2007; Mou et al., 2008; Cano-Monreal et al., 2009). EBV and MCMV infections can induce phosphorylation of at least lamin A/C (Muranyi et al., 2002; Lee et al., 2008). Lamina disruption by lamin phosphorylation is a conserved trait of virus infection by virus or host cell kinases. The cellular isomerase Pin1-induced conformational change of lamins may represent the molecular trigger contributing to the lamina disassembly upon herpes virus infection (Milbradt et al., 2016). Research found that the virus protein ɣ1134.5 could induce phosphorylation and reorganization of lamin A/C by activating PKC, leading to the disintegration of the nuclear lamina in host cells and promote HSV-1 replication (Wu et al., 2016). Similarly, during circovirus infection progression, p32, as the key regulator of porcine circovirus type 2 (PCV2) nuclear egress, can form a complex with the viral capsid protein, increasing the PKC activity and resulting in the rearrangement of the nuclear lamina and facilitate viral nuclear egress *via* lamin A/C phosphorylation (Wang et al., 2019). It has been proved phosphorylation is also essential for neutrophil extracellular trap (NET) formation to protect against infection. Li et al. reported that PKC mediates the nuclear envelope rupture by phosphorylating lamin B1 at S395, S405, and S408 to release chromatin for NET formation in neutrophils (Li et al., 2020).

### Phosphorylation and Development

Apart from the aforementioned functions, lamin phosphorylation is also found to participate in the budding process of large ribonucleoprotein particle (RNP) granules harboring synaptic protein transcripts to exit the nucleus through the inner and the outer nuclear membranes during synapse development, in which it is involved in phosphorylation of A-type lamins (Speese et al., 2012). Furthermore, it has been confirmed that abnormal phosphorylation of lamins was associated with some neurodegenerative diseases, including Alzheimer’s disease (AD). For example, a study found nuclear lamina dispersion occurred due to the direct phosphorylation of lamin A (Ser392) and lamin B1 (Ser393) by Cdk5 in AD primary neurons and animal models, leading to significant neurotoxicity (Chang et al., 2011).

In addition, inhibition of lamin phosphorylation might also lead to pathogenesis during skin development. As is known, nuclear degradation is a critical stage in keratinocyte terminal differentiation and the formation of the cornified envelope, and retention of the nuclear material is a common histological change in many skin diseases, particularly in atopic dermatitis and psoriasis. It has been proved that DNA degradation processes are prevented in the absence of AKT1 phosphorylating lamin A/C for degradation, leading to parakeratosis and changes in epidermal differentiation (Naeem et al., 2015).

The latest research study reported that mitotic polo-like kinase (PLK-1) phosphorylates the lamin LMN-1 to promote the timely lamina disassembly and follow with the merging of the parental genomes into a single nucleus after mitosis in *C. elegans* (Velez-Aguilera et al., 2020), indicating phosphorylation of lamins might be also critical for embryo development.

## Acetylation and the Stability of Lamins

Acetylation is another PTM of lamins, and its function might associate with nuclear periphery stability, cell cycle progression, and DNA repair, as well as nuclear architecture and genome integrity (Karoutas et al., 2019; Murray-Nerger et al., 2021). Acetylation sites of the lamin A/C reside mostly within its coiled-coil rod domain (K97, K108, K114, K270, K311, and K378), and some are at the NLS (K417) and the IgG-fold domain (K450 and K470) (Karoutas and Akhtar, 2021). A recent study showed that loss of the lysine acetyltransferase MOF or its associated NSL-complex members KANSL2 or KANSL3 led to a stochastic accumulation of nuclear abnormalities (Karoutas et al., 2019). They found lamin A/C was an acetylation target of MOF, and the loss of acetylation was accompanied by an increase in S392 phosphorylation of lamin A/C, as a consequence of the increased solubility of lamins, defective phosphorylation dynamics, and impaired nuclear stability.

Moreover, it has been thought that lamin acetylation at the nuclear periphery could prevent capsid nuclear egress to protect against virus production. For example, Murray–Nerger et al. reported that LMNB1 K134 acetylation acted as a host response to suppress human cytomegalovirus (HCMV) production by functionally stabilizing the nuclear periphery (Murray et al., 2018), and their new laboratory findings revealed that LMNB1 K134 acetylation, as a molecular toggle, controlled nuclear periphery stability, cell cycle progression, and DNA repair. It can inhibit lamina disruption during herpes simplex virus type 1 (HSV-1) infection, thereby aborting virus production (Murray-Nerger et al., 2021).

## SUMOylation: A Conserved Post-Translational Modification

Lamins can also be modified with small ubiquitin-like modifiers (SUMOs), known as SUMOylation. The SUMO family contains four conserved 10 kDa proteins, which are SUMO1, SUMO2, SUMO3, and SUMO4, in which SUMO1, 2, and 3 are ubiquitously expressed in all eukaryotic cells, while SUMO4 is a newly found member with unique distribution, which has only been detected in renal, immune and pancreatic cells, and placenta until now (Wang and She, 2008; Chen et al., 2014; Baczyk et al., 2017). They can covalently and reversibly be attached to lysine residues on target proteins (Gareau and Lima, 2010) under the effects of three critical enzymes: SUMO E1 (the heterodimer SAE1 and SAE2), SUMO E2-conjugating enzyme (UBE2I/UBC9), and several E3 ligases (Sekhri et al., 2015; Mattoscio et al., 2017). As reported, the enzymes responsible for SUMO conjugation are located mainly in the nucleus (Gareau and Lima, 2010). A yeast two-hybrid screen found that there was an interaction between lamin A and UBC9, suggesting lamin A could be a target protein of SUMO modification (Zhong et al., 2005). The analysis of the amino acid sequence of lamin A revealed it had the SUMOylation consensus sequence CKXE (MKEE) surrounding lysine 201 in the rod-containing domain (Zhang and Sarge, 2008). Emerging evidence showed that mature lamin A might be SUMOylated at sites in the rod domain by SUMO2 (Zhang and Sarge, 2008) or by SUMO1 and SUMO3 in the tail domain (Galisson et al., 2011; Simon and Wilson, 2013).

SUMOylation involves regulating localization, function, interactions, and stability of target proteins, and influence nuclear import/export, transcription, apoptosis, and cell cycle regulation (Geiss-Friedlander and Melchior, 2007). The SUMOylation of lamins has been reported to be associated with cell cycle progression. Moriuchi et al. have demonstrated interactions between SUMO-interacting motif 3 (SIM3) of lamin A and a putative SUMO2-modified protein, which played an important role in the reorganization of the nuclear lamina at the end of mitosis. Furthermore, a study found that nuclear DNA leakage activates nucleophagy through UBC9-mediated SUMOylation of lamin A/C, leading to degradation of nuclear components including lamin A/C and leaked nuclear DNA (Li et al., 2019), which suggested that lamin SUMOylation might play a role in the degradation of abnormal lamina structures. A few studies have implicated laminopathy mutants could alter lamin SUMOylation. For instance, a research of the Zhang group showed that SUMOylation could regulate lamin A function at lysine 221, and it was lost in lamin A mutants associated with familial cardiomyopathies (Zhang and Sarge, 2008). Boudreau et al. revealed that laminopathy mutants resulted in the mislocalization of SUMO1 both *in vitro* and *in vivo*, suggesting that the mutant lamin A/C altered the dynamics of SUMO1, and thus, misregulation of SUMOylation may be contributing to disease progression in laminopathies (Boudreau et al., 2012).

## Other Post-Translational Modifications: Methylation, Ubiquitination, and O-GlcNAcylation

In addition to the aforementioned PTMs, there are various other PTMs that have been reported in lamins with different cellular and physiological functions. As we have mentioned earlier, mutations in lamin A or ZMPSTE24 cause the premature aging diseases HGPS an RD due to premature lamin A. The processing of mature lamin A required not only farnesylation but also carboxyl methylation of its C-terminal CAAX motif. In addition, it has been reported that C-terminal isoprenylation and carboxyl methylation of lamins increased their hydrophobicity, thus promoting their interaction with relevant inner nuclear membrane proteins (Chelsky et al., 1989; Vorburger et al., 1989; Kitten and Nigg, 1991), and carboxyl methylation of lamin B might also enable it more resistant to proteolysis by interleukin converting enzyme (ICE)-like proteases (e.g., caspases), thereby preventing chromatin condensation and DNA fragmentation culminating in apoptotic cell death (Kowluru, 2000). Moreover, Kim et al. (2011a) found that lamin A/C contained an arginine methylation site by MALDI-TOF analysis and then revealed that lamin A/C was arginine-methylated by PRMT during muscle fusion, with potential implications in muscle development. Depletion of intracellular s-adenosylmethionine (SAM), a key co-substrate of methyl group transfer reactions, damaged the carboxymethylation and maturation of the nuclear lamina, thereby suppressing neurogenic differentiation (Hwang et al., 2021).

It has been reported lamins can also undergo ubiquitination in lamin mutation cells. Khanna et al. (2018) found that RING finger-containing E3 ubiquitin ligase (RNF123) was transcriptionally upregulated in cells expressing rod domain lamin A mutations and then determined that RNF123 could mediate the ubiquitination of these proteins and caused the proteasomal degradation of pRb, LAP2a, and lamin B1, indicating that RNF123-mediated ubiquitination of lamin-binding proteins may contribute to disease-causing mechanisms in laminopathies by depletion of key nuclear proteins and defects in cell cycle kinetics. Another research established HECW2 as an E3 ubiquitin ligase for PCNA and lamin B1 which regulated their levels in laminopathic cells (Krishnamoorthy et al., 2018), suggesting that the interplay among HECW2, lamin A, PCNA, and lamin B1 determines their respective homeostatic levels in the cell, and dysregulation of these interactions may contribute to the pathogenicity of laminopathies. Smurf2 is also reported to be related to ubiquitination of lamin A not proteasomal degradation but its lysosomal degradation (Borroni et al., 2018).

Simon et al. (2018) reported that lamin A could be β-O-linked N-acetylglucosamine-(O-GlcNAc)-modified in human hepatoma (Huh7) cells and in the mouse liver. O-linked glycosylation is the enzymatic addition of O-GlcNAc by the enzyme O-GlcNAc transferase (OGT) to protein Ser/Thr residues (Hart et al., 2011). They also showed robust O-GlcNAcylation of recombinant mature lamin A tails (residues 385–646) with purified OGT enzyme *in vitro* but with no detectable modification of lamin B1, lamin C, or ∆50 (progerin) tails. Then, they identified 11 O-GlcNAc sites in a “sweet spot” (601–645) unique to lamin A with up to seven sugars per peptide by mass spectrometry, and deletion of ∆50 and ∆35 (596–664) reduced O-GlcNAcylation, suggesting residues deleted in progeria are required for substrate recognition and/or modification by OGT *in vitro*.

## Lamin Post-Translational Modifications: Promising Biomarkers and Targets of Laminopathies

**FIGURE 3:**
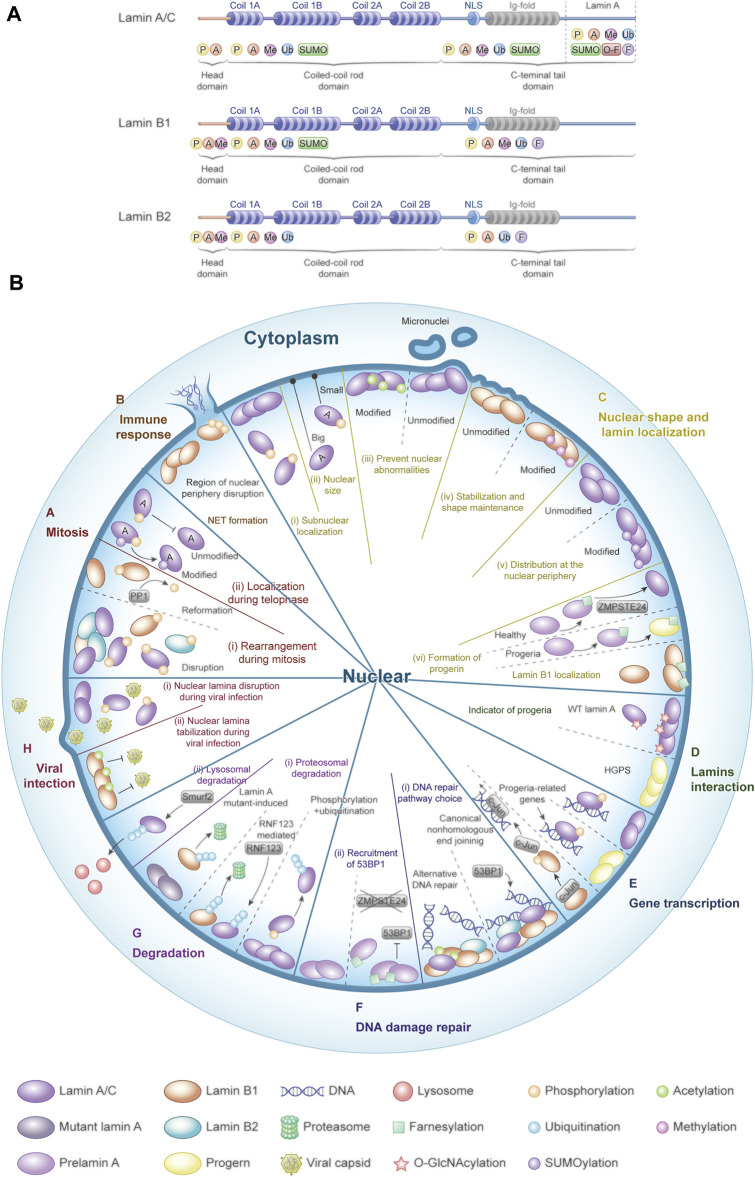
Post-translational modifications of lamins and their multifunctional roles. **(A)** Lamins undergo various PTMs in different domains. **(B)** Landscape of post-translational modifications and functions. PTMs of lamins are involved in mitosis, viral infection and immune response, nuclear shape and lamin localization, lamin interaction, gene transcription, and DNA damage repair, as well as lamin degradation (modified from Murray-Nerger et al., 2021).

The aforementioned PTMs of lamins play critical roles in a variety of cell processes and pathological alteration of some human diseases (Figure 3), especially laminopathies. Preventing post-translation processing to progerin is the main area to target treatment of laminopathies; it kindles the interest in the possibility of lamin PTMs for drug development achieving therapeutic interventions due to the relationship between different PTMs of lamins and laminopathies, as described in this study.

**FIGURE 4:**
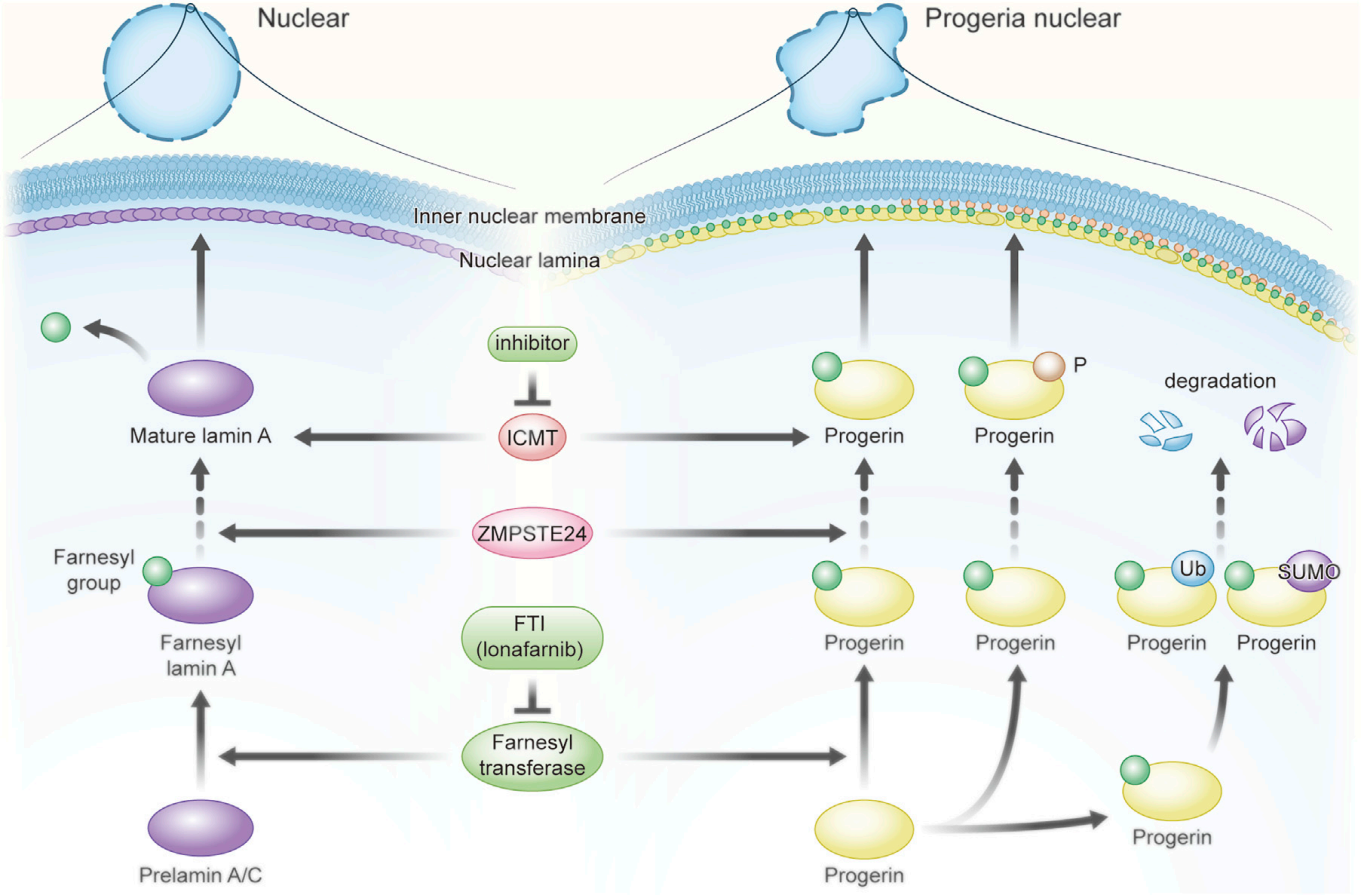
Targeting PTMs of defective lamin A to improve Hutchinson–Gilford progeria syndrome (HGPS). Farnesyltransferase inhibitors (FTI) show promising therapeutic effects due to the reduction of progerin accumulation. The orally active FTI lonafarnib (Zokinvy™) has been approved by the FDA to reduce the risk of mortality in HGPS and for the treatment of processing-deficient progeroid laminopathies in patients ≥12 months of age with a body surface area of ≥0.39 m^2^. Isoprenylcysteine carboxyl methyltransferase (ICMT), phosphorylation, SUMOylation, and ubiquitination of farnesylated prelamin A might also be potential targets for treatment.

As shown in Figure 4, mutated prelamin A fails to be clipped off its farnesylated tail, and accumulation of permanent farnesylated progerin leads to the HGPS. Based on this, inhibitors of farnesyltransferase might be used therapeutically. For example, farnesyltransferase inhibitors (FTIs) might be applied for reducing the accumulation of lots of progerin proteins in the HGPS cells. The fibroblasts from mice and mouse model experiments suggested the FTI could reverse the nuclear shape abnormalities in fibroblasts from the mouse with laminopathies (Yang et al., 2005); cultured cells from human subjects with HGPS also showed nuclear morphological abnormalities, which are reversed by inhibitors of protein farnesylation (Wang et al., 2010). In addition, mouse model experiments also found that FTIs improved some aspects of the disease and increased life expectancy, even though it did not completely halt or reverse the disease process (Smallwood and Shackleton, 2010). The aforementioned research encourages the further investigation of FTIs as a potential treatment for children with HGPS. Two trials were carried out in Europe and USA, in which they used FTIs to prevent farnesylation of lamin A in an attempt to ameliorate or reverse the disease process in HGPS children (Kieran et al., 2007). Another clinical trial also reported that children with HGPS seemed to show a positive response to FTI and lonafarnib treatment (Gordon et al., 2012). It is inspiring that the orally active farnesyltransferase inhibitor lonafarnib (Zokinvy™) has been approved by the FDA to reduce the risk of mortality in HGPS and for the treatment of processing-deficient progeroid laminopathies in patients ≥12 months of age with a body surface area of ≥0.39 m^2^. The clinical results showed that it could prevent farnesylation of lamin A, decrease vascular stiffness, and extend the survival of HGPS patients (Dhillon, 2021; Misteli, 2021). In addition to FTIs, a study showed that the isoprenylcysteine carboxyl methyltransferase (ICMT) inhibitor increased AKT-mTOR signaling and proliferation and delayed senescence in human HGPS fibroblasts, suggesting targeting ICMT might be useful for treating prelamin A-associated progeroid disorders (Ibrahim et al., 2013). As we discussed earlier, Ser22, Y45, and Y481 phosphorylation of farnesylated prelamin A is associated with laminopathies (Ikegami et al., 2020; Lin et al., 2020; Liu and Ikegami, 2020); they might be promising therapeutic targets. Alterations of nuclear morphology and stability are common features in laminopathies, while the acetylation of lamins promotes the nuclear periphery stability. Karoutas et al. found the loss of MOF contributed to abnormal nuclear architecture and genome integrity because its target site, Ser392 of lamin A/C, was phosphorylated (Karoutas et al., 2019). In addition, UBC9-mediated SUMOylation and ubiquitination are reported to participate in degradation of defective lamina structures and some key nuclear proteins individually, involving in disease-causing mechanisms in laminopathies (Zhang and Sarge, 2008; Boudreau et al., 2012; Khanna et al., 2018; Li et al., 2019).

Furthermore, some PTMs of lamins are expected to be clinical diagnostic biomarkers for certain types of laminopathy. For example, Ser458 phosphorylation was only found in lamin A mutants associated with myopathy (Cho et al., 2018). Another PTM, O-GlcNAcylation, was only found in mature lamin A but not in lamin B1, lamin C, or ∆50 (progerin) tails (Hart et al., 2011). Examination of the presence or absence of the aforementioned modification could be used in diagnosing the laminopathies.

## Conclusion and Future Perspective

Lamins are critical for nuclear architecture and function; they are involved in numerous nuclear processes, including chromosomal architecture, peripheral epigenetic landscapes, and eventually gene expression profiles. Diverse PTMs have been identified on lamins and have revealed to regulate the lamin functions in both normal and pathological conditions. PTMs are precisely regulated in a variety of normal physiological processes and specific functions, in which PTMs are altered to adjust the lamin localization, interaction, even epigenetic landscapes, and gene regulatory networks responding to stimuli during various processes. With respect to changes of lamin PTMs in human diseases, such as gene mutations, the PTMs may be caused by those diseases or may participate in the pathogenesis, aggravation, or rescue of the disease, independent or dependent of diseases. Identification of various PTMs and their changes will help us understand the fundamental nuclear biology and their regulation in both biological and clinical contexts and provide therapeutic ways for intervention.

In the future, there are several crucial fields of lamin PTMs that remain to be further explored. The abnormal alterations of PTMs are associated with the nuclear stability and might be eventually involved in lots of pathological states such as laminopathies, cancer, viral infection, and normal or pathological aging. So the first is to investigate the potential changes of lamin PTMs in some human diseases using large-scale proteomic data, experimentally validate their functions, and compare the different PTMs between normal and disease groups for potential biomarkers. The second is to define the key enzymatic machinery during these PTMs and their regulated mechanism. The third is to investigate the crosstalk among these kinds of PTMs, especially the same residue with more than one PTM site. The fourth is to explore whether other protein translational modifications have not been reported in the lamins. Last but not the least is to assess the suitability of PTMs as potential therapeutic targets in diagnosis and treatment.
